# E-cigarettes to Augment Stop Smoking In-person Support and Treatment With Varenicline (E-ASSIST): A Pragmatic Randomized Controlled Trial

**DOI:** 10.1093/ntr/ntac149

**Published:** 2022-06-23

**Authors:** Harry Tattan-Birch, Loren Kock, Jamie Brown, Emma Beard, Linda Bauld, Robert West, Lion Shahab

**Affiliations:** Department of Behavioural Science and Health, University College London, London, UK; SPECTRUM Consortium, UK; Department of Behavioural Science and Health, University College London, London, UK; SPECTRUM Consortium, UK; Department of Behavioural Science and Health, University College London, London, UK; SPECTRUM Consortium, UK; Department of Behavioural Science and Health, University College London, London, UK; SPECTRUM Consortium, UK; SPECTRUM Consortium, UK; Usher Institute, College of Medicine, University of Edinburgh, Edinburgh, UK; Department of Behavioural Science and Health, University College London, London, UK; SPECTRUM Consortium, UK; Department of Behavioural Science and Health, University College London, London, UK; SPECTRUM Consortium, UK

## Abstract

**Aim:**

To examine whether, in adults receiving behavioral support, offering e-cigarettes together with varenicline helps more people stop smoking cigarettes than varenicline alone.

**Methods:**

A two-group, parallel arm, pragmatic randomized controlled trial was conducted in six English stop smoking services from 2019–2020. Adults enrolled onto a 12-week programme of in-person one-to-one behavioral smoking cessation support (*N*  =  92) were randomized to receive either (1) a nicotine e-cigarette starter kit alongside varenicline or (2) varenicline alone. The primary outcome was biochemically verified abstinence from cigarette smoking between weeks 9-to-12 post quit date, with those lost to follow-up considered not abstinent. The trial was stopped early due to COVID-19 restrictions and a varenicline recall (92/1266 participants used).

**Results:**

Nine-to-12-week smoking abstinence rates were 47.9% (23/48) in the e-cigarette-varenicline group compared with 31.8% (14/44) in the varenicline-only group, a 51% increase in abstinence among those offered e-cigarettes; however, the confidence interval (CI) was wide, including the possibility of no difference (risk ratio [RR] = 1.51, 95% CI = 0.91–2.64). The e-cigarette-varenicline group had 43% lower hazards of relapse from continuous abstinence than the varenicline-only group (hazards ratio [HR] = 0.57, 95% CI =  0.34–0.96). Attendance for 12 weeks was higher in the e-cigarette-varenicline than varenicline-only group (54.2% vs. 36.4%; RR = 1.49, 95% CI = 0.95–2.47), but similar proportions of participants in both groups used varenicline daily for ≥8 weeks after quitting (22.9% versus 22.7%; RR = 1.01, 95% CI = 0.47–2.20). Estimates were too imprecise to determine how adverse events differed by group.

**Conclusion:**

Tentative evidence suggests that offering e-cigarettes alongside varenicline to people receiving behavioral support may be more effective for smoking cessation than varenicline alone.

**Implications:**

Offering e-cigarettes to people quitting smoking with varenicline may help them remain abstinent from cigarettes, but the evidence is tentative because our sample size was smaller than planned—caused by Coronavirus Disease 2019 (COVID-19) restrictions and a manufacturing recall. This meant our effect estimates were imprecise, and additional evidence is needed to confirm that providing e-cigarettes and varenicline together helps more people remain abstinent than varenicline alone.

## Introduction

Rates of cigarette smoking are declining in many high-income countries,^1^ in part due to the availability of treatments that help people stop smoking.^2^ Varenicline—a partial nicotinic acetylcholine receptor agonist—is one of the most effective treatments, especially when paired with behavioral support.^3^ However, even with varenicline, fewer than one-in-five people remain abstinent from smoking for a year or more after quitting,^4^ so there remains a need to find more effective options. Electronic cigarettes (e-cigarettes)^5^ have become a popular method of quitting cigarette smoking in England, used in a third of quit attempts.^6^ E-cigarettes can deliver similar amounts of nicotine as cigarettes but, by avoiding tobacco combustion, expose users to much lower levels of toxicants.^7–9^ Offering electronic cigarettes (“e-cigarettes”) alongside varenicline and behavioral support may help people maintain abstinence from smoking conventional cigarettes.

The rationale for providing e-cigarettes alongside varenicline is two-fold. First, e-cigarettes mimic the sensory and behavioral aspects of smoking that contribute to dependence,^10^ something which is not provided by varenicline. Second, the pharmacological effects of varenicline may be enhanced by providing additional nicotine. The main target of varenicline is the α4β2 subtype of nicotinic acetylcholine receptors, an important mediator of nicotine dependence.^11^ However, other functionally important subtypes (e.g., α6β2) may not be fully saturated by varenicline, allowing nicotine from other sources to bind to increase receptor activation. Moreover, varenicline does not fully stop the dopaminergic effects of smoking, and additional nicotine may bind to other receptors important to dependence that varenicline does not affect.^12^ It may also be that the pharmacokinetics of varenicline and alternate nicotine delivery devices complement one another to provide a more favorable agonistic effect on receptors.^13^

Observational data from English stop smoking services show that people who use nicotine e-cigarettes, varenicline, and behavioral support together are more successful in their attempts to quit smoking than those using any other treatment.^14^ Moreover there is trial evidence that combination therapy of nicotine replacement therapy (NRT) and varenicline is safe and well-tolerated and may increase abstinence rates compared with varenicline alone,^12^ particularly for more dependent smokers,^15^ and compared with NRT alone in alcohol-dependent smokers.^16^ However there are no trial data on combination therapy of e-cigarettes with varenicline. E-cigarettes may offer an additional advantage over NRT not only because they more closely mimic cigarettes, but also because they have been found to be more effective nicotine delivery devices, increasing abstinence rates compared with NRT.^17,18^ One trial in New Zealand had aimed to evaluate the effectiveness and safety of combining varenicline with nicotine e-cigarettes for smoking cessation among those with mental health illnesses, but it was stopped due to difficulties in using participants.^19^ As far as we are aware, there are no studies taking place investigating combination therapy of varenicline with e-cigarettes against varenicline alone in routine stop smoking services. If found to be effective in an RCT, this could become a new gold standard treatment for smoking cessation.

This pragmatic trial aims to answer the following question: In adults receiving one-to-one behavioral support at English stop smoking services, does offering nicotine e-cigarette starter kits together with varenicline increase cigarette abstinence rates compared with varenicline alone?

We also aim to examine how offering e-cigarettes to clients affects attendance at stop smoking services, adherence to varenicline, and e-cigarette use. Moreover, a qualitative process evaluation aims to explore the acceptability of offering e-cigarettes alongside varenicline at services, as well as barriers and enablers to using them.

## Methods

### Design

This is a two-group, parallel arm, pragmatic randomized controlled trial. It was conducted between April 2019 and March 2020 in stop smoking services in England, which are free to access for smokers trying to quit. Fifteen services were approached to take part in the study, of which eight (53%) agreed to participate and six (40%) started enrollment. Reasons for not participating included lack of staff capacity, incompatible models of service delivery, and concerns about e-cigarettes (Supplementary Table S1).

Services used participants and delivered the intervention during one-to-one in-person counseling sessions with trained stop smoking advisors. Participants were randomized (1:1 ratio in blocks of 6 or 8 participants, stratified by service) using a computer-generated random sequence with allocation concealed within opaque envelopes. Due to the nature of the intervention, participants and advisors could not be blinded to treatment assignment.

Ethical approval was granted by both University College London (8323/003) and the NHS Health Research Authority (19/LO/0239). The study was overseen by both a trial steering and a data monitoring committee. The trial protocol and analysis plan were registered prior to participant used (ISRCTN16931827) and were peer-reviewed as a registered report at *N and TR*. Updates were approved by the data monitoring committee prior to unblinding or analysis of data. These updates added secondary analyses of continuous abstinence and respiratory symptoms, as well as sensitivity analyses for the primary outcome (Supplementary Table S2). The original and updated protocols are available online, alongside a summary of changes (https://osf.io/vm4g3/).

### Procedures

In their first session, smokers were asked to set a target quit date, usually within one to 4 weeks, and advisors used a checklist to assess eligibility for inclusion in the trial. Cigarette smokers were eligible if they were proficient in English, were not pregnant or breast feeding, opted to use varenicline, were willing to try e-cigarettes, and had not regularly used e-cigarettes in the past 6 months.

Advisors gave eligible smokers trial information and a consent form. After smokers provided written informed consent, advisors recorded baseline characteristics, took an exhaled carbon monoxide (CO) reading, and opened opaque envelopes to reveal whether smokers were randomized to the e-cigarette-varenicline group or the varenicline-only group.

This study was designed to avoid interfering with standard service protocols. Following existing practice, participants in both randomized groups were prescribed varenicline and given behavioral support during regular in-person sessions with their advisor. They were offered weekly or fortnightly support until 12 weeks after their quit date. Behavioral support aimed to minimize participants’ motivation to smoke, maximize their motivation to remain abstinent, and guide their use of pharmacotherapy—as described in detail elsewhere.^20^ During each session, advisors recorded smoking status, exhaled CO, adherence, adverse events, and respiratory symptoms using existing software (QuitManager or PharmOutcomes).

The COVID-19 pandemic led all in-person sessions to be stopped after March 2020. Advisors remotely followed up with those (*n* = 5) who had yet to complete their final 12-week appointment, using CO-monitors that had been posted to participants to verify abstinence.

#### Varenicline-Only Group

Participants were prescribed the standard 12-week course of varenicline, starting approximately 2 weeks prior to their target quit date. They were advised to take one 0.5 mg pill daily for the first 3 days, then two 0.5 mg pills daily for days 4 to 7, and finally two 1 mg pills daily for the remaining 11 weeks. As this was a pragmatic trial, participants were not asked to avoid using e-cigarettes.

#### E-cigarette-Varenicline Group

These participants also received a standard 12-week course of varenicline described above. In addition, they were given an e-cigarette starter kit prior to their quit date. The starter kit contained an Aspire PockeX e-cigarette (as used in previous trials),^17,21^ e-liquid to last for approximately 4 weeks, and an information booklet about e-cigarettes (available here: https://osf.io/59adw/). Participants could choose a total of eight 10 ml e-liquid bottles (from Aspire or Totally Wicked) in any combination from a selection of three flavors (fruit, menthol, and tobacco) and three nicotine concentrations (6, 12, and 18 mg/ml). Participants were encouraged to buy further bottles from local vape shops. Advisors gave participants brief in-person advice about how to use e-cigarettes and asked them to try the e-cigarette during the session. As this pragmatic trial aimed to test the effect of offering—not using—an e-cigarette, participants were asked but not required to use them.

### Measures

At every session after quitting, participants were asked whether they had smoked cigarettes since their previous session, with exhaled CO-readings of below 10 ppm used to verify cigarette abstinence.^22^ They were also asked, since their last session, how frequently they had used varenicline or e-cigarettes and whether they had experienced specific adverse events (sleep disturbance, nausea, and throat/mouth irritation) or respiratory symptoms (phlegm, cough, shortness of breath, and wheezing). Advisors were required to report serious adverse events to the trial team, but none occurred throughout the trial. Further, details about questionnaire items are available in Supplementary Table S3.

Nine-to-12-week smoking abstinence was the primary outcome, with participants considered abstinent if they (1) reported not smoking cigarettes between weeks 9 and 12 after their quit date and (2) gave a CO-reading below 10 ppm at week 12 or later. Participants with missing data for the primary outcome were assumed not to be abstinent.

Secondary abstinence outcomes included two-to-four-week smoking abstinence (defined as above) and length of continuous abstinence before relapse. The latter outcome was not included in the original protocol but was added to the updated protocol and registered prior to data analysis (https://osf.io/vm4g3/). It was measured as the number of weeks, from the quit date onwards, that each participant remained continuously abstinent from smoking before relapsing.

Attendance was assessed using two outcomes. Firstly, whether or not a participant continued attending sessions until at least 12 weeks after the quit date. Secondly, the number of sessions, of a possible four, a participant attended in their first 4 weeks after quitting.

Two outcomes assessed adherence to varenicline. Firstly, whether or not participants reported using varenicline daily for at least 1 week after their quit date and, secondly, whether they used varenicline daily until at least 8-weeks after quitting. The latter allows up to 4 weeks of varenicline use prior to quitting. E-cigarette outcomes were daily use for at least 1 week after the quit date and daily use at every session attended after quitting.

Time to first experiencing each adverse event and respiratory symptom were recorded for each participant.

### Analysis

Data analyses were conducted by the trial statistician with blinding to treatment assignments using R version 4.1.3.^23^ Anonymized data and analysis code are openly available (https://osf.io/vdngh/). The primary and other binary outcomes were reported as risk ratios (RR) with 95% confidence intervals (95% CIs). Analyses of binary smoking abstinence outcomes followed the intention-to-treat principle, where all those with missing follow-up data were treated as having relapsed (0% abstinent).

In sensitivity analyses for the primary outcome, RRs were calculated with a range of different assumed abstinence rates in those lost to follow-up (e.g., 10%, 20%, 30%, and 40%).

Moreover, for length of continuous abstinence from quit date onwards, the hazard ratio (HR) for relapse was estimated using a Cox proportional-hazards model. A HR of less than one means that participants in the e-cigarette-varenicline group had a lower rate of relapse and thus remained abstinent for longer than those in the varenicline-only group. Participants who were lost to follow-up were assumed to have relapsed in the week after the final stop smoking session they attended where CO-measurements were taken. Participants who were still abstinent at week 12 were considered censored after this time.

Unplanned sensitivity analyses for the primary outcome adjusted for e-cigarette nonadherence (i.e., people in the e-cigarette-varenicline group who did not try e-cigarettes) and contamination (i.e., people in the varenicline-only group who tried e-cigarettes), using a method described by Cuzick et al..^24^ This provides an estimate of the effect of trying e-cigarettes (daily use for at least a week) among cooperators: Individuals who would try e-cigarettes if they were assigned to the e-cigarette-varenicline group, but would not try them if assigned to the varenicline-only group.^25^

Cox models were also used to estimate the HR for time to first experiencing each adverse event and respiratory symptom. These were reported alongside the incidence rate for each randomized group (i.e., the number of people who reported an event divided by the person-weeks-at-risk), with the incidence rate ratio (IRR) estimated using a log-rate model. For these analyses, participants were considered censored after the final week they attended a follow-up session (maximum 12 weeks post quit date).

### Sample Size and Early Stopping

As described in the original study protocol (https://osf.io/vxw8r/), previous literature suggested an expected risk ratio of 1.26 for our primary outcome.^12,14^ It was determined that a sample of 633 participants per group would provide at least 90% power to detect this effect size in a two-tailed analysis.

Restrictions introduced in response to the COVID-19 pandemic caused services to move sessions online, which meant advisors could not provide e-cigarettes to participants or take in-person CO-readings. This led the trial to be paused in March 2020, before the target number of participants had been used (92/1266). We started planning amendments to the procedures to allow the trial to continue remotely, including behavioral support being given via telephone or video call and cigarette abstinence being verified remotely using saliva anabasine and anatabine. These plans were halted when, in July 2021, Pfizer recalled Champix (the only form of varenicline available in England) due to levels of N-nitroso-varenicline that were higher than considered acceptable by the European Medicines Agency.^26^ In agreement with the funder, Pfizer, the trial was stopped in November 2021.

### Process Evaluation

Quantitative process evaluation included summaries of attendance at stop smoking services, varenicline adherence, and e-cigarettes adherence and/or contamination.

Qualitative process evaluation involved semi-structured interviews using a flexible topic guide (https://osf.io/2pgz4/) carried out with ten participants from the e-cigarette-varenicline group who had been followed up until at least 4 weeks after their quit date. Interview transcripts were analyzed in two stages, using a combination of deductive and inductive thematic framework analysis. Firstly, themes surrounding the acceptability of services providing e-cigarettes alongside varenicline were classified under the theoretical framework of acceptability (TFA).^27^ Then, barriers and enablers to using e-cigarettes for smoking cessation under the COM-B model were identified.^28^ More details about the process evaluation and are provided elsewhere.^29^

## Results

### Participants

**Figure 1.**
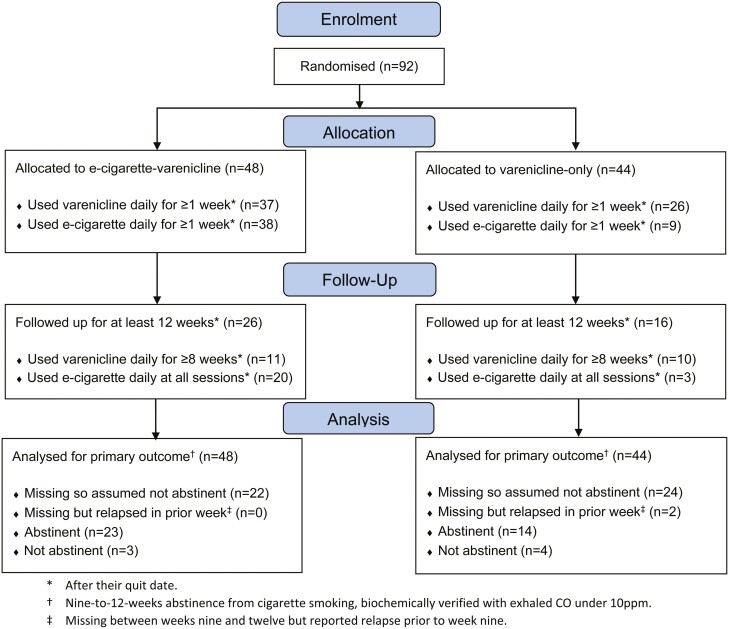
CONSORT flow diagram. A software issue meant it was only possible to determine the number of participants who were both eligible for and willing to take part in the trial, not the total number who were approached. Reasons for loss to follow-up were not recorded due to the pragmatic nature of the trial. *After their quit date. †Nine-to-12-weeks abstinence from cigarette smoking, biochemically verified with exhaled CO under 10 ppm. ‡Missing between weeks 9 and 12 but reported relapse prior to week 9.

Of the 92 cigarette smokers randomized at stop smoking services between April 2019 and March 2020, 48 were assigned to the e-cigarette-varenicline group and 44 to the varenicline-only group. Participants had a mean age of 43.9 (*SD* = 13.1), 51% (*n *= 47) were women, 79% (*n* = 73) were white, and 29% (*n* = 27) had routine or manual occupations (Table 1). Table 1 shows that participants in both randomized groups had similar baseline characteristics. Of those randomized, 46% (*n* = 42) attended follow-up sessions for at least 12 weeks after their quit date (Figure 1).

**Table 1.** Baseline Characteristics^*^

	E-cigarette	Control	Combined
*N*	48	44	92
Age	43.8 ± 12.1	44.0 ± 14.2	43.9 ± 13.1
Gender			
Woman	52% (25)	50% (22)	51% (47)
Man	48% (23)	50% (22)	49% (45)
Ethnicity			
White	79% (38)	80% (35)	79% (73)
Black or Asian	17% (8)	11% (5)	14% (13)
Other or mixed	4% (2)	9% (4)	7% (6)
Occupation			
Managerial or professional	40% (19)	39% (17)	39% (36)
Routine or manual	27% (13)	32% (14)	29% (27)
Other^†^	33% (16)	30% (13)	32% (29)
Free prescription			
Not reported	71% (34)	66% (29)	68% (63)
Yes	29% (14)	34% (15)	32% (29)
Anxious or depressed			
No	77% (37)	68% (30)	73% (67)
Yes	24% (11)	32% (14)	27% (25)
Cigarettes per day^‡^			
≤10	15% (3)	30% (7)	23% (10)
11–20	45% (9)	48% (11)	47% (20)
21–30	30% (6)	22% (5)	26% (11)
≥31	10% (2)	0% (0)	5% (2)

Age presented as mean ± standard deviation. All other characteristics summarized as % (*n*).

Includes people who are retired, unemployed, or home carers.

Only recorded for 43 participants: 20 in the e-cigarette-varenicline (e-cigarette) group and 23 in the varenicline-only (control) group.

### Smoking Abstinence

#### Primary — Nine-to-12-week Abstinence

Nine-to-12-week abstinence rates were 47.9% (*n* = 23) in the e-cigarette-varenicline group compared with 31.8% (n = 14) in the varenicline-only group. This equates to a 1.51-fold increase in abstinence rates in those offered e-cigarettes; however, the confidence interval was wide and included the possibility of no difference (RR 1.51, 95% CI .91–2.64). Bayes factors are shown in Supplementary Table S4. Results were similar when including quits that were self-reported but not biochemically verified (52.1% versus 34.1%; RR 1.53, 95% CI .95–2.60).

Supplementary Table S5 shows sensitivity analyses that relax the assumption that all participants missing for the follow-up had relapsed. These show that the higher the percentage of missing participants who were abstinent, the smaller the estimated effect size (e.g., RR 1.38 if 20% of missing participants were abstinent).

#### Secondary — Two-to-four-week Abstinence

Two-to-four-week abstinence rates were 1.37 times higher in the e-cigarette-varenicline than varenicline-only group, but the confidence interval was compatible with effects ranging from just under no difference to 2.01 times higher rates in those offered e-cigarettes (68.8% versus 50.0%; RR 1.37, 95% CI .98–2.01).

#### Secondary — Relapse From Continuous Abstinence

**Figure 2.**
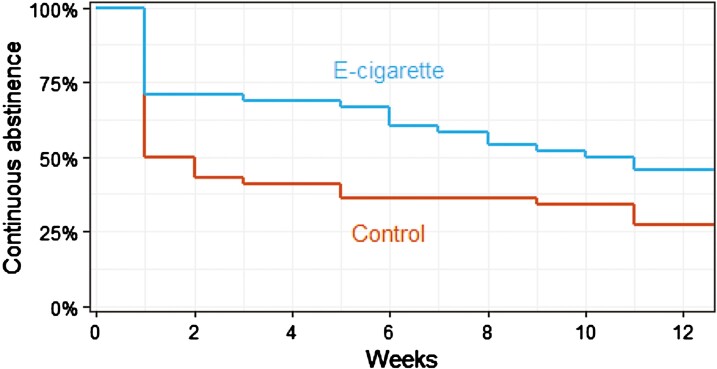
Kaplan–Meier plot showing the percentage of participants continuously abstinent (CO < 10 ppm) from cigarette smoking at each week after their quit date. Participants who were lost to follow-up were assumed to have relapsed in the week after the final session they attended.

The e-cigarette-varenicline group had a 43% lower (instantaneous) rate of relapse from continuous cigarette abstinence than those in the varenicline-only group (Cox model; HR 0.57, 95% CI .34–0.96). Figure 2 shows a Kaplan-Meier plot for the length of time each participant remained continuously abstinent from cigarettes before relapsing. Note that these analyses were not included in the original protocol but were added to the updated protocol which was registered prior to data analysis.

### Safety

#### Adverse Events

Overall, 59.8% (*n* = 55) of participants experienced at least one adverse event between their quit date and final session. Sleep disturbance was reported by 44.6% (*n* = 41) of participants, nausea by 34.8% (*n* = 32), and throat or mouth irritation by 27.2% (*n* = 25). Comparisons of event incidence rates and hazard ratios between the e-cigarette-varenicline and varenicline-only group are shown in Table 2. These estimates were too imprecise to determine the size or direction of differences between groups (e.g., any adverse event; HR 0.69, 95% CI .40–1.20). Risks of adverse events among those followed-up for at least 12 weeks are shown in Supplmentary Table S6. No serious adverse events were reported in either group.

**Table 2.** Incidence of Adverse Event and Respiratory Symptoms

	Group^*^	Events	Weeks^†^	Rate^‡^	IRR (95%CI)^‡^	HR (95%CI)^‡^
*Adverse events*						
Any	Control	24	144	1.67	Ref	Ref
	E-cigarette	31	193	1.61	0.96 (0.57–1.66)	0.69 (0.40–1.20)
Sleep disturbance	Control	21	163	1.29	Ref	Ref
	E-cigarette	20	283	0.71	0.55 (0.30–1.02)	0.64 (0.34–1.20)
Nausea	Control	14	209	0.67	Ref	Ref
	E-cigarette	18	261	0.69	1.03 (0.51–2.11)	0.84 (0.41–1.72)
Throat/mouth irritation	Control	7	244	0.29	Ref	Ref
	E-cigarette	18	310	0.58	2.02 (0.88–5.21)	1.11 (0.45–2.74)
*Respiratory symptoms*						
Any	Control	19	164	1.16	Ref	Ref
	E-cigarette	25	258	0.97	0.84 (0.46–1.54)	1.05 (0.57–1.92)
Phlegm	Control	13	210	0.62	Ref	Ref
	E-cigarette	20	293	0.68	1.10 (0.55–2.27)	0.75 (0.37–1.53)
Cough	Control	14	200	0.70	Ref	Ref
	E-cigarette	17	301	0.56	0.81 (0.40–1.66)	1.49 (0.72–3.08)
Shortness of breath	Control	8	225	0.36	Ref	Ref
	E-cigarette	12	332	0.36	1.02 (0.42–2.59)	1.22 (0.48–3.10)
Wheezing	Control	6	249	0.24	Ref	Ref
	E-cigarette	7	381	0.18	0.76 (0.25–2.37)	0.85 (0.26–2.82)

There were 44 participants in the varenicline-only (control) group and 48 in the e-cigarette varenicline (e-cigarette) group.

Total person-weeks at risk of first event. For each person, this is the number of weeks from the quit date until they either experienced the event/symptom, were lost to follow-up, or completed the study (12 weeks post quit).

Incidence rate calculated per 10 person-weeks. Incidence rate ratios (IRR) and corresponding 95% confidence intervals (95%CI) estimated using log-linear rate models. Hazards ratios (HR) and corresponding 95% CIs estimated using Cox proportional-hazards models. Schoenfeld tests found some evidence for nonproportional hazards for throat/mouth irritation (*p* = .046) and cough (*p* = .032), but all other outcomes were compatible with proportionality (*p* > .31).

#### Respiratory Symptoms

Respiratory symptoms were reported by 47.8% (*n* = 44) of participants at least once between their quit date and the final session they attended. Phlegm was reported by 35.9% (*n* = 33) of participants, cough by 33.7% (*n* = 31), shortness of breath by 21.7% (*n* = 20), and wheezing by 14.1% (*n* = 13). Table 2 shows that rates of respiratory symptoms were similar in the e-cigarette-varenicline and varenicline-only group (e.g., any symptom; HR 1.05, 95% CI .57–1.92), but confidence intervals included the possibility of meaningful differences between groups.

### Process Evaluation: Quantitative Data

#### Attendance

Of the 92 participants randomized, 45.7% (*n* = 42) continued attending stop smoking service sessions for at least 12 weeks after their quit date. Attendance for 12 weeks was 54.2% (*n* = 26) in the e-cigarette-varenicline group compared with 36.4% (*n* = 16) in the varenicline-only group (RR 1.49, 95% CI .95–2.47). On average, participants in the e-cigarette-varenicline group attended 3.1 out of a possible four sessions in the first four weeks after quitting, while those in the varenicline-only group attended 2.8 sessions (proportional-odds model; OR 1.69, 95% CI .93–2.45).

#### Varenicline Adherence

In the e-cigarette-varenicline group, 77.1% (*n* = 37) of participants used varenicline daily for at least 1 week after their quit date, compared with 59.1% (*n* = 26) in the varenicline-only group (RR 1.30, 95% CI .99–1.79). Daily varenicline use for at least eight weeks after quitting was reported by 22.9% (*n* = 11) of participants in the e-cigarette-varenicline group and 22.7% (*n* = 10) in the varenicline-only group (RR 1.01, 95% CI .47–2.20).

#### E-cigarette Adherence and Contamination

In the e-cigarette-varenicline group, 79.2% (*n* = 38) used e-cigarettes daily for at least 1 week after their quit date, and 41.7% (*n* = 20) reported daily use at every session they attended after quitting. There was some contamination: 20.5% (*n* = 9) of participants in the varenicline-only group used e-cigarettes daily for at least 1 week after their quit date, and 6.8% (*n* = 3) reported daily use at every session they attended after quitting.

In an unplanned analysis of the primary outcome that adjusted for nonadherence (i.e., being assigned to try e-cigarettes but not doing so) and contamination (i.e., being assigned to the control group but trying e-cigarettes), trying e-cigarettes was estimated to increase nine-to-12-week abstinence by 2.66 times (RR 2.66, 96% CI 1.17–6.05).^24^

### Process Evaluation: Qualitative Data

#### Acceptability

Themes surrounding the acceptability of providing e-cigarettes alongside varenicline at stop smoking services were identified from semi-structured interviews with ten participants in the e-cigarette-varenicline group (Supplementary Table S7 and https://osf.io/2pgz4/). Participants perceived the intervention package as complementary, with varenicline reducing urges to smoke and the e-cigarette replacing the habit of smoking. However, some were concerned that e-cigarettes may “replace one addiction with another” and, thus, there were mixed opinions about whether services should provide e-cigarette.

#### Barriers and Enablers

Barriers and enablers to using e-cigarettes for smoking cessation are shown in Supplementary Table S8. Enablers included the perception that e-cigarettes replace the habit of smoking and offer a “back-up” to varenicline and behavioral support when participants are most at risk of relapse. E-cigarettes were also described as cheaper than cigarettes and could be used in more situations than smoking. Some participants reported the harshness of puffing of e-cigarettes as a barrier to using them, especially early in their quit attempt.

## Discussion

### Summary

Our study provides tentative evidence that, among people receiving one-to-one behavioral support, offering e-cigarettes alongside varenicline may be more effective for cigarette smoking cessation than varenicline alone. The evidence is tentative because our sample size was smaller than planned—caused by COVID-19 and a manufacturing recall—which meant our effect estimates were imprecise (highly compatible with 9% lower to 164% greater nine-to-12-week abstinence rates in those given e-cigarettes). More data are needed to confirm whether providing e-cigarettes and varenicline together helps more people remain abstinent than varenicline alone.

### Comparison With Prior Literature

Nonetheless, our study adds to a wider literature on the effects of offering alternative nicotine products alongside varenicline. Our results closely align with a previous meta-analysis finding the 50% higher odds of cigarette abstinence in those given NRT alongside varenicline than varenicline alone (OR 1.50, 95%CI 1.14–1.97).^12^ However, another recent study showed that adding nicotine patches to varenicline had little effect on abstinence rates (OR 0.99, 95% CI .87–1.12).^30^ It is possible that fast-acting nicotine products—including gums, sprays, and e-cigarettes—are better at helping varenicline users remain abstinent, as they can satisfy momentary urges for nicotine.^31^ Moreover, the behavior and sensory experience of using an e-cigarette is similar to that of smoking a cigarette, which could make e-cigarette more effective for smoking cessation than other nicotine products.

### Interpretation

We found that most of the difference in relapse between groups occurred within the first week after quitting. Three quarters of participants in the e-cigarette group remained abstinent for at least 1 week compared with just half of those in the varenicline-only group (Figure 2). This could be explained by e-cigarettes helping people overcome the intense urges to smoke most people experience in the first few days after quitting.^32–34^ However, it is possible that some people entered the study because they wanted a free e-cigarette. In learning they had been randomised to the control group, they may have bought an e-cigarette elsewhere and stopped attending sessions. Because the primary analysis assumed that people with missing follow-up data had relapsed to smoking, this could lead us to overestimate differences in abstinence rates between groups (as shown in sensitivity analyses in Supplementary Table S1).

Conversely, other factors could have led us to underestimate any effect using e-cigarettes had on abstinence. For instance, there was some nonadherence with only 79% of participants in the e-cigarette-varenicline group trying their e-cigarette for at least a week after quitting. There was also some contamination, with 20% of those in the varenicline-only group trying e-cigarettes. In unplanned sensitivity analyses adjusting for this nonadherence and contamination, our estimate for the effect of e-cigarettes on increasing 9-to-12-week abstinence tripled from 51% to 166%.

Secondary analyses indicated that e-cigarettes help people remain continuously abstinent from cigarettes for longer, with data most compatible with 43% lower instantaneous rate of relapse in the e-cigarette-varenicline than varenicline-only group. This analysis rested on the assumption that people who were lost to follow-up had relapsed in the week after the final session they attended. However, because lost to follow-up was greater in the varenicline-only group, this could have biased results in favor of e-cigarettes.

### Process Evaluation

In interviews, participants reported that they viewed the e-cigarettes, varenicline, and behavioral support to be acceptable and complementary, but some were concerned about continued nicotine use and the harshness of vaping. These concerns may be alleviated by providing information around the relative harms of smoking versus vaping,^35^ giving advice about titrating inhalation to avoid harshness, or providing products that are less harsh to inhale—such as those using lower pH nicotine salts e-liquid.^36,37^ Our results align with previous studies showing that people who are worried about the addictiveness of nicotine use too little NRT, which stops them from benefiting from it.^38^ These worries may be especially pronounced for e-cigarettes, both because long-term use is more common with e-cigarettes than NRT^17^ and because negative perceptions about the harms of e-cigarettes have become increasingly prevalent over time.^39–41^

### Strengths and Limitations

The study benefits from using randomized assignment, which provides internal validity (exchangeability), and a pragmatic design within stop smoking services that guarantees ecological validity (given that this is the setting where such an intervention would likely be implemented). However, there were several limitations.

First, clients could not be blinded to their assigned group. This is an inherent limitation of many smoking cessation trials. We partially militated against it by using objective biochemical measures (CO-readings) to verify abstinence from cigarette smoking, which reduces the risk of outcome assessment being biased by assessors knowing which group participants were assigned to. Second, services only followed up with clients for 12 weeks after quitting, and because this is a pragmatic trial, we did not ask them to extend this period. This meant abstinence was measured for less than the 6 months recommended by Russell Standard guidelines.^42^ Third, just under half of the participants continued attending services until their final 12 week follow-up session, with 50% greater lost to follow-up in the e-cigarette-varenicline than varenicline-only group. Our primary analysis assumed those with missing follow-up data had relapsed, which is likely a reasonable assumption as people tend to only continue attending services if they remain abstinent. Nonetheless, in sensitivity analyses (Supplementary Table S4) we quantitatively assessed how certain violations of this assumption would affect results.^43^ We did not model assumed abstinence rates for those lost to follow-up being higher in the control than for the intervention group. This would have been the most conservative assumption but unlikely in the context of our trial where both arms were receiving similarly intensive in-person support. Fourth, a fifth of those in the varenicline-only group used e-cigarettes while a fifth of those in the e-cigarette-varenicline group did not. This contamination and nonadherence would dilute any effect of using e-cigarettes on abstinence, but we accounted for this in a sensitivity analysis. Fifth, we compared combination treatment with e-cigarettes and varenicline to varenicline alone among smokers receiving intensive behavioral support. Our results cannot inform us about the relative effectiveness of e-cigarettes alone versus varenicline alone, and they cannot be generalized to settings where smokers receive minimal support. Finally, trial enrollment was stopped early due to the COVID-19 pandemic and recall of varenicline by Pfizer, which meant we lacked a sufficiently large sample to precisely estimate effects of treatment.

## Conclusion

In conclusion, we found tentative evidence that, among people receiving one-to-one behavioral support, providing e-cigarettes alongside varenicline may be more effective than offering varenicline alone. However, estimates were imprecise due to the lower than planned sample size; for the primary outcome, anything from 9% lower to 164% higher abstinence rates remained highly compatible with the data (at the 95% level). More data are needed to clarify the effect of adding e-cigarettes to smoking cessation treatment with varenicline.

## Supplementary Material

A Contributorship Form detailing each author’s specific involvement with this content, as well as any supplementary data, are available online at https://academic.oup.com/ntr.

ntac149_suppl_Supplementary_Material

## Data Availability

Anonymized study data are available here (https://osf.io/t8qu9/).
