# Supplementary material for: E-cigarettes to Augment Stop Smoking In-person Support and Treatment With Varenicline (E-ASSIST): A Pragmatic Randomized Controlled Trial
Source: Nicotine Tob Res. 2022 Jun 23;25(3):395–403. doi: 10.1093/ntr/ntac149 (PMC9384384; doi:10.1093/ntr/ntac149)
Supplement: ntac149_suppl_Supplementary_Material [file ntac149_suppl_supplementary_material.docx]

**Supplementary Tables and Figures**

Table S1: Reasons for stop smoking services not participating in the trial.

| **Service*** | **Reason for not participating** |
| --- | --- |
| **1** | No response after initial contact |
| **2** | Delivery of services through pharmacies incompatible with trial procedures |
| **3** | Service closed down before the trial started |
| **4** | Lack of staff capacity |
| **5** | Delivery of services through general practices incompatible with trial procedures |
| **6** | No response after initial contact |
| **7** | Perceived lack of evidence on e-cigarette harms and use for smoking cessation |

* Names of services are excluded for data protection purposes

| **Planned and registered before data collection** | **Planned/updated and registered before data analysis** | **Unplanned** |
| --- | --- | --- |
| Analyses of smoking-related outcomes following intention-to-treat principle where those lost to follow-up are treated as smokers | Sensitivity analyses for the primary outcome where risk ratios were calculated with a range of different assumed abstinence rates in those lost to follow-up | Sensitivity analysis for the primary outcome adjusting for e-cigarette non-adherence and contamination |
| Bayes factors for the primary outcome | Hazard ratio (HR) for relapse from continuous abstinence estimated using a Cox model |  |
| Treatment adherence (varenicline adherence and e-cigarette use) across groups | HR and incidence rate ratio for adverse events and respiratory symptoms in the e-cigarette versus control group |  |
| Interviews with ten participants in e-cigarette arm on acceptability and barriers and enablers to participation | Attendance at stop smoking services across groups |  |

Table S2: Summary of planned and unplanned analyses.*

* Reasons for updates to the protocol are discussed in detail online (<https://osf.io/vm4g3/>).

Table S3: Questions added to data collection system at services

| **Construct assessed** | **Question added** |
| --- | --- |
| Trial eligibility | Eligible participant agreed to participate in UCL trial (Y/N) |
| Trial arm allocation | If Y selected above, enter treatment allocation (E-cigarette/Control) |
| Varenicline adherence | How often have used Varenicline since last session? |
|  | N/A |
|  | Daily |
|  | Weekly |
|  | Less Than Weekly |
|  | Did not use |
| E-cigarette usage | If the e-cigarette checkbox is checked a further two fields will appear: |
|  | Date device given |
|  | Date field with calendar helper (will retain date from previous session if already populated) |
|  | How often have used e-cigarette since last session? |
|  | Not Applicable |
|  | Daily |
|  | Weekly |
|  | Less Than Weekly |
|  | Did not use |
| Adverse reactions | Since the last visit/contact, has the participant experienced any of the following adverse reactions: |
|  | Nausea (Y/N) |
|  | Sleep disturbance (Y/N) |
|  | Throat or mouth irritation (Y/N) |
| Respiratory symptoms | Since the last visit/contact, has the participant experienced any of the following respiratory symptoms: |
|  | Shortness of breath (Y/N) |
|  | Wheezing (Y/N) |
|  | Cough (Y/N) |
|  | Phlegm (Y/N) |
| Mental health | Please select one of the below that describes the participant’s health TODAY: |
|  | Not anxious or depressed |
|  | Slightly anxious or depressed |
|  | Moderately anxious or depressed |
|  | Severely anxious or depressed |
|  | Extremely anxious or depressed |

Table S4: Bayes factors calculated for the primary outcome, nine-to-12 weeks cigarette abstinence.

| **Observed RR* (95% CI)** | **RR under H0** | **RR under H1‡** | **Bayes factor†** |
| --- | --- | --- | --- |
| 1.51 (0.91-2.64) | 1.00 | 0.50 | 0.17 |
| 1.51 (0.91-2.64) | 1.00 | 0.66 | 0.27 |
| 1.51 (0.91-2.64) | 1.00 | 0.80 | 0.44 |
| 1.51 (0.91-2.64) | 1.00 | 1.25 | 1.91 |
| 1.51 (0.91-2.64) | 1.00 | 1.50 | 2.04 |
| 1.51 (0.91-2.64) | 1.00 | 2.00 | 1.69 |

* Risk ratios (RR) and corresponding 95% confidence intervals (95% CI) estimated from log-linear risk models.

‡ H1, the alternative hypothesis for log(RR), was modelled as a half normal distribution with a mode at zero and a standard deviation equal to log of the RR listed in this column.

† Bayes factor from online calculator (<http://www.bayesfactor.info>). Bayes factors above 1 indicate greater support for alternative hypothesis (H1) than the null hypothesis (H0), while those below 1 indicate greater support for H0 than H1.

Table S5: Nine-to-12-week cigarette CO-verified abstinence rates when relaxing the assumption that participants with missing follow-up data at week 12 had relapsed (i.e., 0% abstinence rate).

| **Imputed abstinence rate in missing*** | **Group** | **Missing / N** | **Abstinence rate (n)†** | **RR†** |
| --- | --- | --- | --- | --- |
| 0% | Control | 28 / 44 | 31.8% (14.0) | Ref |
|  | E-cigarette | 22 / 48 | 47.9% (23.0) | 1.51 |
| 10% | Control | 28 / 44 | 36.8% (16.2) | Ref |
|  | E-cigarette | 22 / 48 | 50.8% (24.4) | 1.38 |
| 20% | Control | 28 / 44 | 41.8% (18.4) | Ref |
|  | E-cigarette | 22 / 48 | 53.8% (25.8) | 1.29 |
| 30% | Control | 28 / 44 | 46.8% (20.6) | Ref |
|  | E-cigarette | 22 / 48 | 56.7% (27.2) | 1.21 |
| 40% | Control | 28 / 44 | 51.8% (22.8) | Ref |
|  | E-cigarette | 22 / 48 | 59.6% (28.6) | 1.15 |

* Imputed abstinence rate among participants who were missing at the 12 weeks post-quit follow-up appointment.

† Estimated percentage and number (n) of people abstinent from cigarette smoking between weeks nine and 12 post-quit, after imputing the abstinence rate in those missing at follow-up. Risk ratio (RR) calculated from these estimates.

Table S6: Adverse event risk among those attending the week 12 follow-up session.

| **Adverse event** | **Group** | **Events*** | **N** | **Risk** | **RR (95%CI)†** |
| --- | --- | --- | --- | --- | --- |
| Any | Control | 12 | 16 | 75.0% | Ref |
|  | E-cigarette | 21 | 26 | 80.8% | 1.08 (0.77-1.51) |
| Sleep disturbance | Control | 11 | 16 | 68.8% | Ref |
|  | E-cigarette | 14 | 26 | 53.8% | 0.78 (0.48-1.27) |
| Nausea | Control | 6 | 16 | 37.5% | Ref |
|  | E-cigarette | 14 | 26 | 53.8% | 1.44 (0.69-2.97) |
| Throat/mouth irritation | Control | 6 | 16 | 37.5% | Ref |
|  | E-cigarette | 13 | 26 | 50.0% | 1.33 (0.64-2.80) |

* Number of participants experiencing at least one event between their quit date and their final follow-up session.

† Risk ratios (RR) and corresponding 95% confidence intervals (95% CI) estimated from log-linear risk models.

Table S7: Summary of findings on acceptability of the intervention.

| **TFA domain** | **Theme** | **Effect on acceptability*** |
| --- | --- | --- |
| Affective attitude | Positive affect for advisor | + |
| Burden | Difficulties with service care pathwa*y* | - |
|  | Side-effects from varenicline | - |
| Ethicality | E-cigarette replaces one addiction with another | - |
|  | Opinions about services providing e-cigarettes | +/- |
| Intervention coherence | Complementary nature of intervention package | + |
| Perceived effectiveness | Varenicline reduces urges to smoke | + |

* + = enhances acceptability; **-** = reduces acceptability; +/- = differing effects on acceptability. See supporting data at <https://osf.io/2pgz4/>.

Table S8: Summary of findings on barriers and enablers to using e-cigarettes for smoking cessation

| **COM-B domain** | **Theme** | **Barrier or Enabler *** |
| --- | --- | --- |
| Automatic motivation | Replacing the habit of smoking | E |
| Reflective motivation | E-cigarette as a back-up in the quit attempt | E |
|  | E-cigarette is a short-term tool to quit smoking | E/M |
| Physical capability | Harshness of puffing | B |
| Physical opportunity | Cost saving | E |
|  | Opportunities to vape | E |
| Social opportunity | Family support to quit smoking | E |

* B = barrier; E = enabler; M = mixed. See supporting data at <https://osf.io/2pgz4/>.
